# Correlation Networks for Identifying Changes in Brain Connectivity during Epileptiform Discharges and Transcranial Magnetic Stimulation

**DOI:** 10.3390/s140712585

**Published:** 2014-07-14

**Authors:** Elsa Siggiridou, Dimitris Kugiumtzis, Vasilios K. Kimiskidis

**Affiliations:** 1 Department of Electrical and Computer Engineering, Aristotle University of Thessaloniki, Thessaloniki 54124, Greece; E-Mail: esingiri@auth.gr; 2 Laboratory of Clinical Neurophysiology, AHEPA Hospital, Medical School, Thessaloniki 54124, Greece; E-Mail: kimiskid@med.auth.gr

**Keywords:** transcranial magnetic stimulation (TMS), electroencephalograms, epileptic discharges, cross-correlation, partial cross-correlation, causality networks

## Abstract

The occurrence of epileptiform discharges (ED) in electroencephalographic (EEG) recordings of patients with epilepsy signifies a change in brain dynamics and particularly brain connectivity. Transcranial magnetic stimulation (TMS) has been recently acknowledged as a non-invasive brain stimulation technique that can be used in focal epilepsy for therapeutic purposes. In this case study, it is investigated whether simple time-domain connectivity measures, namely cross-correlation and partial cross-correlation, can detect alterations in the connectivity structure estimated from selected EEG channels before and during ED, as well as how this changes with the application of TMS. The correlation for each channel pair is computed on non-overlapping windows of 1 s duration forming weighted networks. Further, binary networks are derived by thresholding or statistical significance tests (parametric and randomization tests). The information for the binary networks is summarized by statistical network measures, such as the average degree and the average path length. Alterations of brain connectivity before, during and after ED with or without TMS are identified by statistical analysis of the network measures at each state.

## 1. Introduction

In multivariate time series analysis the interaction of the observed variables can simply be assessed on the basis of the linear correlation between two variables *X* and *Y*, namely cross-correlation and partial cross-correlation conditioning on the other observed variables. The correlation may involve a time lag *τ*, *i.e.*, correlation of *X*(*t*) and *Y*(*t* + *τ*), and in conjunction with pre-whitening of the time series of *X* and *Y* (elimination of autocorrelation or drifts), it can estimate time directed interactions and serve as a simple Granger causality measure [1]. Further, correlation networks can be formed having as nodes the observed variables and connections determined by the estimated cross-correlation or partial cross-correlation. In particular, the connections can be undirected when *τ* = 0 or directed when *τ* ≠ 0, and they can be weighted given directly by the correlation or binary given by the decision for significant (one) or non-significant (zero) correlation [2]. The analysis of multivariate time series by means of correlation networks is a rather new approach with applications in many fields ranging from economics [3] to neurophysiology [4,5].

In this work we apply the aforementioned approach to investigate the impact of transcranial magnetic stimulation (TMS) on the brain connectivity structure when applied during epileptiform discharges (ED). In recent years, TMS has emerged as a promising non-invasive brain stimulation technique with therapeutic potential in focal epilepsy [6–9]. Recent studies have shown that EDs are connected with changes in the dynamics of the brain and possible alterations in connectivity between inter-connected areas of the brain [10]. In this study, we investigate whether correlation networks derived from multi-channel scalp EEG can detect changes in brain connectivity before and during the ED, as well as after the ED when it terminates naturally or due to the TMS administration.

The structure of the paper is as follows: in Section 2, the methodology is presented giving a brief account of correlation networks, discussing in particular the derivation of binary networks from statistical significance test, and presenting the TMS-EEG protocol. In Section 3, the application of the framework of correlation networks to the TMS-EEG is presented and conclusions are given in Section 4.

## 2. Methodology

We assume an observed multi-variate time series ${\left\{{\text{y}}_{t}\right\}}_{t=1}^{N}={\left\{y_{1,t},y_{2,t},\ldots ,y_{n,t}\right\}}_{t=1}^{N}$, such as an EEG segment of *n* channels and duration of *T* s, where *N* = *Tf*_s_, and *f*_s_ is the sampling frequency. Each component time series ${\left\{y_{i,t}\right\}}_{t=1}^{N}$ is processed to remove autocorrelation. This is achieved by fitting to ${\left\{y_{i,t}\right\}}_{t=1}^{N}$ an autoregressive model of an order *p*, AR(*p*), and taking the residual time series ${\left\{x_{i,t}\right\}}_{t=1}^{N}$, a procedure commonly known as pre-whitening [11]. The order *p* can be fixed or adapted by some model order estimation criterion, such as the Akaike information criterion or AIC. Pre-whitening is equally effective in removing non-stationary effects, such as drifts, provided an appropriate order *p* is selected. The correlation analysis is thus performed on the pre-whitened multi-variate time series ${\left\{{\text{x}}_{t}\right\}}_{t=1}^{N}={\left\{x_{1,t},x_{2,t},\ldots ,x_{n,t}\right\}}_{t=1}^{N}$. Let also the observed vector variable and its components at a time *t* be denoted as ***X****_t_* ={*X*_1,_*_t_*, *X*_2,_*_t_*, …, *X_n_*_,_*_t_*} or simply ***X*** ={*X*_1_, *X*_2_, …, *X_n_*}.

The cross-covariance matrix is:

(1) $$C(\tau )=1/(N-\tau )\sum_{t=1}^{N-\tau }({\text{x}}_{t}-\bar{\text{x}}){\left({\text{x}}_{t+\tau }-\bar{\text{x}}\right)}^{\text{T}}$$

for lag *τ*, where ***x̄*** is the mean vector. The element *c_ji_*(*τ*) of *C*(*τ*) denotes the covariance of*X_i_*_,_*_t_* and *X_j_*_,_*_t+τ_*, *i.e.*, the cross-covariance of *X_i_* and *X_j_* at lag *τ*, and the cross-correlation at lag *τ* is:

(2) $$r_{ji}(\tau )\equiv r_{X_{j}X_{i}}(\tau )=c_{ji}(\tau )/\sqrt{c_{ii}(\tau )c_{jj}(\tau )}$$

The *r_ji_*(*τ*) for *τ* > 0 indicates the linear correlation of *X_i_* and *X_j_* at a future time *τ*, and this can be interpreted as Granger causality from *X_i_* to *X_j_*, and *vice versa* for *τ* < 0 [1].

Cross-correlation does not distinguish direct correlation between *X_i_* and *X_j_* from indirect correlation, *i.e.*, because of each one's correlation with another variable *X_k_*. To exclude indirect correlation of *X_i_* and *X_j_* with respect to all other components of ***X***, stacked in the variable set *Z_n_*_−2_ of cardinality *n*−2, the partial cross-correlation can be used, denoted *ϕ_ji_*(*τ*) ≡ *r_XjXi_*_|__*Z*_*n*−2__(*τ*), which estimates the correlation of *X_i_* and *X_j_* that is not already contained in the correlation of *X_i_* or *X_j_* with any of the variables in *Z_n_*_−2_. To derive *ϕ_ji_*(*τ*), firstly, the residuals of the linear regression of *X_i,t_* on all the variables of *Z_n_*_−2_ at the same time *t*, and the residuals of the linear regression of *X_j,t_*_+_*_τ_* on all the variables of *Z_n_*_−2_ at time *t* are obtained, and then *ϕ_ji_*(*τ*) is the correlation coefficient of the residuals of *X_i_*_,_*_t_* and *X_j_*_,_*_t_*_+_*_τ_* [12].

### 2.1. Statistical Significance Test of Correlation

To draw a binary network from the correlation matrix or the partial correlation matrix one needs to decide whether the estimated cross-correlation *r_ji_*(*τ*) or partial cross-correlation *ϕ_ji_*(*τ*) for a pair of variables *X_i_* and *X_j_* is significant or not. Denoting here *r* any of *r_ji_*(*τ*) or *ϕ_ji_*(*τ*) forsimplifying the notation, the parametric test for the null hypothesis of zero correlation uses the test statistic (e.g., see Chapter 10.3 in[2]):

(3) $$t=r\sqrt{\left(N-2)/(1-r^{2}\right)}$$

which follows the Student distribution with *N*−2 degrees of freedom, assuming that the pair (*X_i_*, *X_j_*) follows bivariate Gaussian distribution. A binary network is derived using the significance test, *i.e.*, when the *p*-value of the test is less than the significance level *a*, a connection is assigned to the pair (*X_i_*, *X_j_*) (the component of the adjacency matrix gets the value one), otherwise there is no connection (zero value), and this method of deriving binary networks is called P-VALUE. To correct for multiple testing, as the same test applies for any *i* and *j* in {1, …, *n*}, we use the correction of the false discovery rate (FDR) [13]. The *p*-values of *m* tests, where *m* = *n*(*n*−1)/2, are set in ascending order *p*_(1)_ ≤ *p*_(2)_ …≤*p*_(_*_m_*_)_. The rejection of the null hypothesis of zero correlation at the significance level *α* is decided for all variable pairs for which the *p*-value of the corresponding test is less than *p*_(_*_k_*_)_, where *p*_(_*_k_*_)_ is thelargest *p*-value for which *p*_(_*_k_*_)_≤*k*·*α*/*m* holds. This method for constructing binary networks is called FDR.

Since the assumption of Gaussian distribution cannot always be fulfilled, we also consider a randomization significance test. The test relies on the generation of time series resampled from the original time series and being consistent with the null hypothesis. We apply here the resampling method of time-shifted surrogates [14]. Each time series ${\left\{x_{j,t}\right\}}_{t=1}^{N}$ is temporally displaced by a random time step *w_j_*, so that the new resampled time series is $\left\{x_{j,t}^{*}\}=\{x_{j,w_{j}+1},x_{j,w_{j}+2},\ldots ,x_{j,N},x_{j,1},\ldots ,x_{j,w_{j}}\right\}$. In this way we destroy possible cross-correlations so that the time series are consistent with the null assumption, but possible autocorrelations and also the marginal distribution of the original time series are preserved.

We calculate the statistic *r* (where *r* is *r_ji_*(*τ*) or *ϕ_ji_*(*τ*)) on the initial pair of time series, call it *r*_0_, and on each of the *M* pairs of time-shifted surrogates (here we use *M*= 1000), denoted *r*_1_,*r*_2_,…,*r_M_*. If *r*_0_ is at the tails of the empirical distribution which is formed from *r*_1_,*r*_2_,…,*r_M_* the null hypothesis is rejected. The rank of *r*_0_ in the ordered list of *M* + 1 values *r*_0_,*r*_1_,*r*_2_,…,*r_M_*, denoted *i*_0_, defines the *p*-value of the randomization test. Concretely, if *i*_0_ < (*M* + 1)/2 then:

(4) $$p=2\frac{i_{0}-0.326}{M+1+0.348}$$

otherwise:

(5) $$p=2\frac{1-(i_{0}-0.326)}{M+1+0.348}$$

where the correction in [15] is used for the empirical cumulative distribution. The binary network derived by the *p*-values of the randomization significance test is called P-VALUE-R. The correction of FDR is applied also to the *p*-values of the randomization tests and this method for constructing binary networks is called FDR-R. The *p*-values from the parametric test and the randomization test, and also the corrections with FDR, are defined for the significance of both *r_ji_*(*τ*) or *ϕ_ji_*(*τ*) at a predefined *α* =0.05.

A simpler approach to determine significance of correlation is to set an arbitrary threshold, here set to 0.1, and this way of constructing binary networks is denoted THRESH. If the value of the correlation between two variables is greater than the value of the threshold then there is connection between them in the network. Such a small threshold was found appropriate because the time series have already undergone pre-whitening and the cross-correlation, along with the autocorrelation, has been reduced.

### 2.2. Network Measures

There are a number of network statistical measures capturing different characteristics of the network structure [16, 17]. Here, we use two such measures defined for each node of a binary network, namely the node degree and the shortest path length. The node degree for a node *i* is the sum of all binary connections between the node *i* and any other node *j*. The shortest path length for node *i* is the average of all the shortest path lengths between node *i* and each other node *j*. Further, the average degree and average shortest path length for the binary network is the average of the node degree and the average of the short path length over all nodes, respectively.

### 2.3. TMS-EEG Protocol

**Figure 1.**
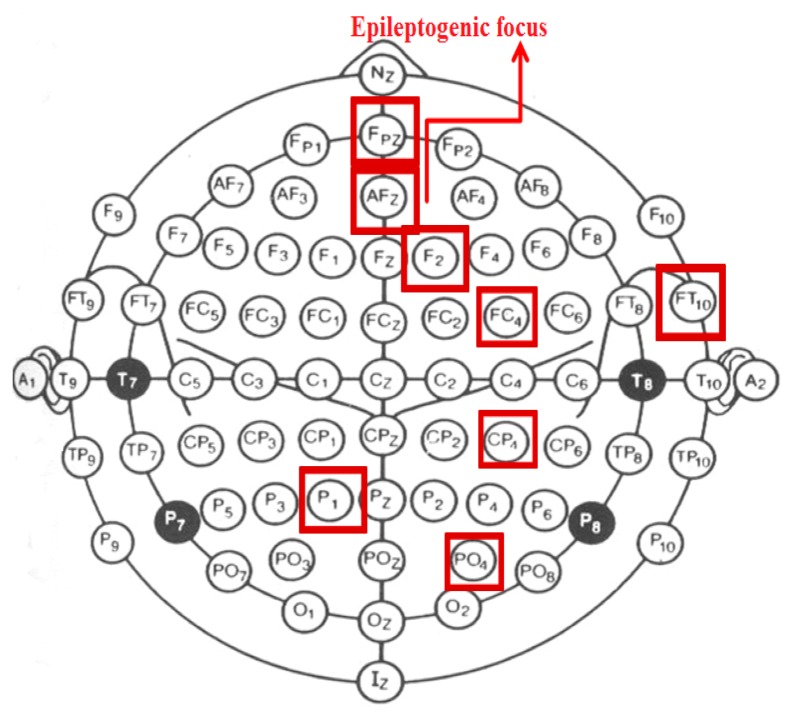
Positions of electrodes on the scalp surface according to 10-10 system. The electrodes of whose the measurements where used in this study are shown in rectangular frames. The label “Epileptogenic focus” indicates the area of the epileptogenic zone.

The study participant gave informed consent for the procedures, which were approved by an institutional review board and performed in accordance with the ethical standards laid down in the 1964 Declaration of Helsinki. The patient was a 38 year old female suffering from drug-resistant, structural frontal lobe epilepsy. Her EEG contained particularly frequent epileptiform discharges that is spikes, sharp waves or spike-wave complexes as well as longer-lasting subclinical electrographic seizure patterns [18]. The epileptogenic zone was located on the frontal convexity on the right and therefore was easily accessible to transcranial stimulation (Figure 1).

TMS-EEG recordings were performed according to recent methodological guidelines [19] in an electrically shielded room. For brain stimulation, we used a repetitive magnetic stimulator (Magstim Rapid, Magstim, Dyfed, Wales) with a circular coil (Magstim type P/N 9784-00) positioned manually over the epileptogenic focus.

The TMS-induced artifact was minimized by employing the minipuncture technique [20]. EEG was recorded with a 60-channel TMS-compatible EEG amplifier (eXimia, Nexstim Ltd., Helsinki, Finland). During acquisition, the reference channel was attached to the left mastoid and the ground electrode was placed on the left zygomatic bone. The EEG signals were band-pass filtered from 0.1 to 500 Hz and sampled with a 1450 Hz sampling frequency and 16-bit precision.

We followed the TMS protocol described in detail in [8]. Briefly, brain stimulation (trains of 1-5 stimuli at 5 Hz and a stimulus intensity corresponding to the lower epileptogenic threshold) was applied at variable treatment latencies after the onset of EDs in an intermittent fashion so that EDs in conjunction with TMS were interleaved with EDs in the absence of TMS. It should be noted that the time periods between successive EDs were in the order of tens of seconds. In view of the short-lived character of TMS-induced perturbations of brain activity it is therefore unlikely that carry-over effects might have acted as a confounder in the analysis.

For the analysis in this study, we chose eight EEG channels, as highlighted in Figure 1. In choosing EEG channels, we were guided by topographical criteria as well as the quality of the recorded signal. With regard to the former, our intention was to sample EEG data primarily from the area of the epileptogenic zone, that is the convexity of the right frontal lobe. In addition, we wanted to include representative channels from the remaining parts of the homologous hemisphere and to a lesser extent from the non-affected left hemisphere. The second critical factor was the quality of the signal meaning that the selected channels had to be consistently free of any biological (*i.e.*, muscle activity, EOG artifacts, *etc.*) or mechanical artifacts.

## 3. Application of Correlation Networks to TMS-EEG

**Figure 2.**
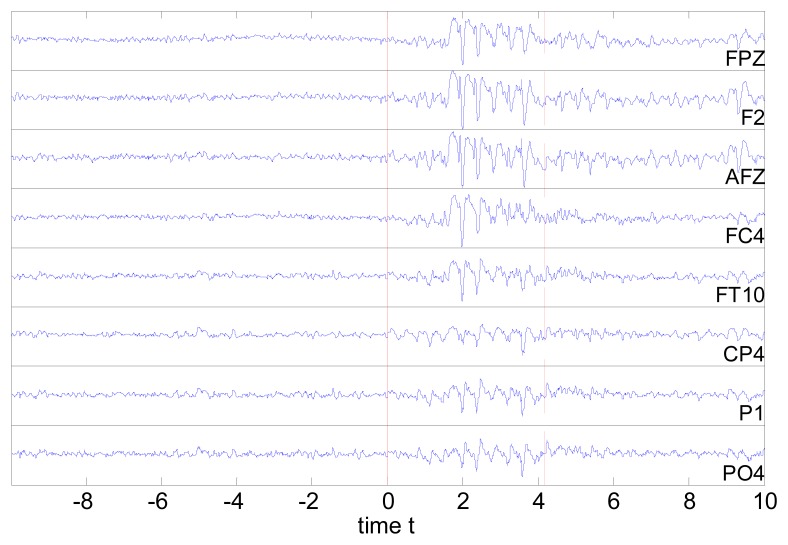
EEG from eight channels as denoted at each panel for an episode with ED starting at zero time point, denoted by the vertical solid line, and TMS administered approximately 4 s later, denoted by the vertical dashed line. The ED terminated about 12 s after the start (not shown).

We analyzed 20 episodes of epileptiform discharges. TMS was applied in 10 of them. In each episode, we picked out the last 10 s of an 8-channel EEG recording prior to the occurrence of ED (denoted preED). For the episodes without TMS administration, we considered also the first 10 s after the start of ED (denoted ED). For each of the two states, 10 non-overlapping windows of 1 s were used (*N* = 1450 observations) and for each window we calculated the cross-correlation and partial cross-correlation matrix for *τ* = 0 and *τ* = 1, which defined the undirected and directed weighted networks, respectively. The network nodes were the eight channels highlighted in Figure 1. Further, we used the five methods for the significance of correlation presented in Section 2.1 to form the respective five binary adjacency matrices and the respective binary networks. For the episodes with TMS administration, in order to address the pure effect of TMS we considered only the 2 first seconds after the onset of brain stimulation, denoted postTMS. We chose a 2-s period as a minimal common denominator of TMS after-effects in view of the varying number of stimuli (1-5) delivered during EDs. To compare with the state of the ED just before the TMS, we considered also the last 2 s before the TMS, denoted preTMS. For preTMS and postTMS, we applied the same procedure but to only two consecutive windows of 1 s. An EEG segment from eight channels is shown in Figure 2 for an episode containing 10 s preED, ED before and after TMS.

**Figure 3.**
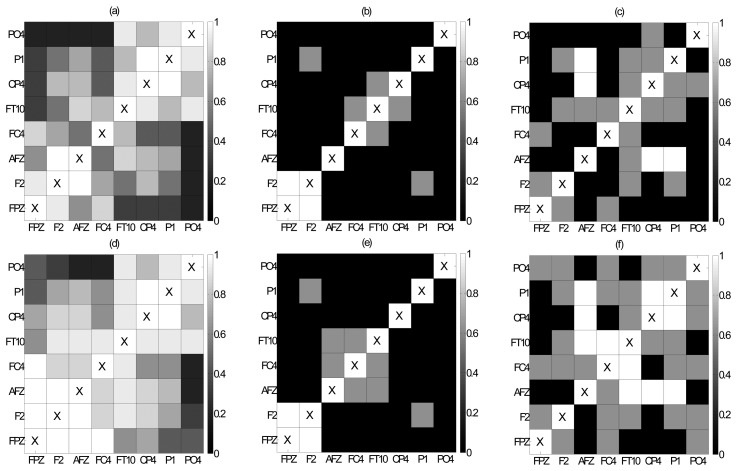
For the episode in Figure 2, the average of the binary connections constructed by the method THRESH and *r_ji_*(0)over 10 binary networks for 10 s of preED in (**a**), and accordingly 2 s of preTMS in (**b**) and postTMS in (**c**). In (**d**–**f**), the same results are displayed but for the method FDR-R and *r_ji_*(0).

The average connectivity structure at each state of an episode (pre-ED and ED for an episode without TMS, and preED, preTMS and postTMS for an episode with TMS) is obtained by the component-wise mean of the adjacency matrices over the corresponding 10 or two windows. For illustration, we discuss in the following the results for the episode shown in Figure 2. In Figure 3, the connectivity structure at preED (10 s before the ED start), preTMS (2 s right before the TMS) and postTMS (2 s right after the TMS) for this episode is shown by color maps regarding the average over all adjacency matrices for windows of 1 s given by the cross-correlation at lag zero, *r_ji_* (0), and the methods THRESH (binary connection when a value is larger than 0.1) and FDR-R (FDR correction to the *p*-values of the randomization test). The average degree for THRESH changes from 3.8 for preED (Figure 3a), to 0.62 for preTMS (Figure 3b) and 1.75 for postTMS (Figure 3c), while for the average shortest path length the corresponding values are 1.33, 0.35 and 1.82. Accordingly, for FDR-R, average degree changes as 5.92 for preED (Figure 3d), 0.75 for preTMS (Figure 3e) and 3.0 for postTMS (Figure 3f), while average path length changes as 1.15, 0.45 and 1.29, respectively. These results on preED, preTMS and postTMS show that the connectivity decreases with the ED and is regained with the TMS. Further, we observe that before the epileptiform discharge there is significant correlation between the nodes that are near the epileptogenic focus (channels FPZ, F2 and AFZ) and in the middle and back of the brain (channels CP4, P1 and PO4, see Figure 3a,d), whilst during the ED and before the administration of TMS there is an overall reduction in the correlation at these areas (Figure 3b,e). With TMS administration we observed that the reduction in the correlation is not so prominent and appears to mitigate the difference between preED and ED state (Figure 3c,f).

**Figure 4.**
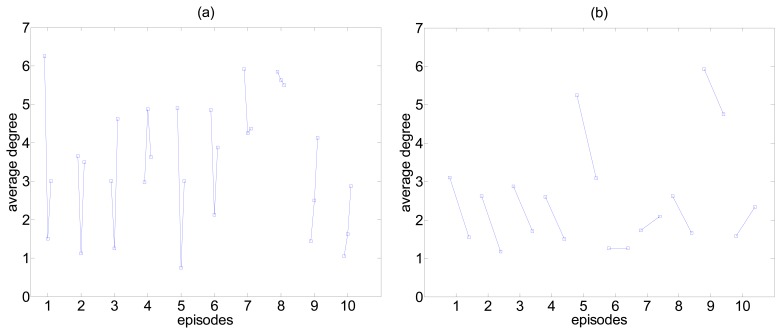
The average degree on binary networks given by the method FDR-R and *r_ji_*(0) for the 10 episodes with TMS (the three values at each episode correspond to preED, preTMS and postTMS) in (**a**) and the 10 episodes without TMS (the two values correspond to preED and ED) in (**b**).

The reduction of correlation with the ED is observed in many episodes but not all. Representative results for the average degree on binary networks given by the method FDR-R and the *r_ji_*(0) are shown in Figure 4 for the 10 episodes with TMS (preED, preTMS, postTMS) and the 10 episodes without TMS (preED, ED). In the majority of cases, TMS tends to change correlation towards the average level of correlation at the preED state, which corresponds to about 4 degrees per node on average. For the 10 episodes without TMS, we observe that in all but three episodes the average degree gets smaller during ED, and this decrease is also observed when TMS is present (comparing preED and preTMS). It is notable that the level of preED is not the same across episodes, which justifies the use of paired comparisons (preED-ED, preED-preTMS and preED-postTMS).

**Figure 5.**
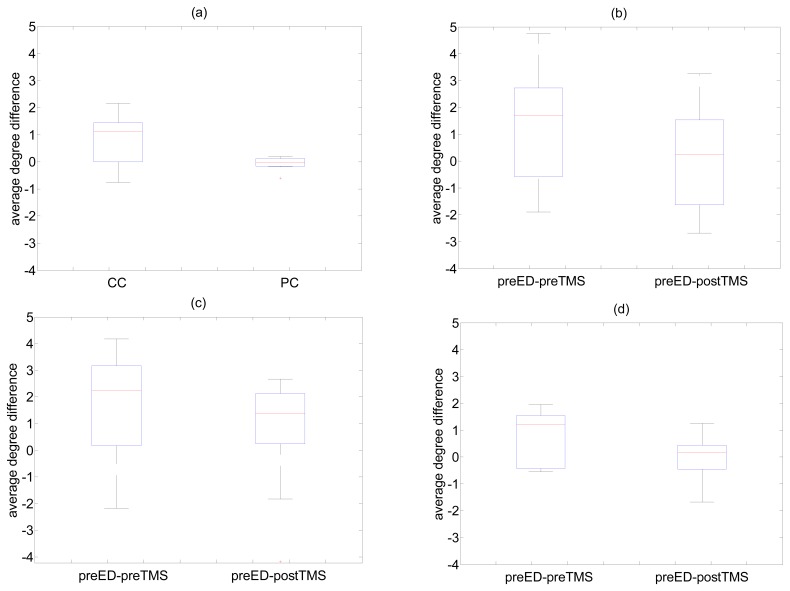
(**a**) Boxplots for the average degree differences of preED-ED for the method FDR-R using *r_ji_*(0) (denoted CC) and *ϕ_ji_*(0)(denoted PC); (**b**) Boxplots for the average degree differences of preED-preTMS and preED-postTMS for the method FDR-R using *r_ji_*(0); (**c**) As in (b) but for the method P-VALUE and *r_ji_*(1); (**d**) As in (b) but for the method FDR and *ϕ_ji_*(0).

Further, the grand average connectivity structure for each state (preED and ED for episodes without TMS, or preED, preTMS and postTMS for episodes with TMS) is obtained by the mean of these adjacency matrices over the 10 episodes without TMS and the 10 episodes with TMS. To evaluate the change of connectivity between preED and ED state, we applied paired samples student test for the equality of mean network measure for ED and preED state, as well as the area under ROC curve (AUROC), where an observation in the sample is taken from the average network measure value computed on the 10 windows of an episode. Non-parametric test (Wilcoxon sign-rank test) gave similar results to student test, and indeed overall there was no evidence of a significant deviation from Gaussian distribution for any of the network measures in the 10 windows. Similarly, the t-test and AUROC were computed for the pairs preED–preTMS and preED–postTMS. The spread of the difference in the average degree values at the states preED and preTMS, preED and postTMS, as well as preED and ED, are shown in the form of boxplots in Figure 5 for different methods. For the case of the difference preED-ED, when this is derived by FDR-R and *r_ji_*(0) it is spread mainly above the zero level, indicating statistically significant decrease of the brain connectivity quantified by the average degree, when going from the preED state to the ED state (Figure 5a). This however does not seem to be established by FDR-R and *ϕ_ji_*(0) as the spread of the average degree difference is around the zero level (Figure 5a). The positive difference using FDR-R and *r_ji_*(0) is also obtained for the pair preED-preTMS, which is essentially the same as preED-ED but preTMS is restricted to 2 s, while after TMS the difference (preED-postTMS) is decreased to the zero level (Figure 5b). Similar differences of preED-preTMS and preED-postTMS are obtained with other methods, as shown in Figures 5c,d for two exemplary cases of different lag and correlation type, namely P-VALUE and *r_ji_*(1), and FDR and *ϕ_ji_*(0), respectively. In all these cases, the difference of preED-preTMS is found statistically significant using the paired t-test (also the Wilcoxon sign rank test), whereas the difference preED-postTMS is not found statistically significant.

The statistical comparison on the paired sample of average degree from 10 episodes using the paired t-test and AUROC shows that the difference of the correlation before and during the ED, *i.e.*, either the pair preED-ED or the pair preED-preTMS, is often found statistically significant giving AUROC values well above 0.5 (the level of no difference). This was found with different methods, and in particular with the zero-lag cross-correlation, as shown in Table 1. On the other hand, the administration of TMS made this difference less significant, and statistically significant difference for the pair preED-postTMS was not found by any combination of method, correlation measure and lag (only marginally with *p* = 0.069 for P-VALUE-R and *ϕ_ji_*(0)). The smallest AUROC values for the difference preED-postTMS were obtained for lag zero, at about the 0.6 level, to be contrasted to the AUROC for the difference preED-preTMS, as well as preED-ED, being at the 0.7 level. We note that the difference for preED-ED is not as large as for preED-preTMS for all combinations of method, correlation measure and lag, e.g., for *r_ji_*(1) the AUROC is smaller for preED-ED than for preED-preTMS for any method. Also, the randomization test for the significance of *ϕ_ji_*(0) and *ϕ_ji_*(1) (methods P-VALUE-R and FDR-R) seems to give smaller differences for preED-preTMS, as well as preED-ED, than the other three methods. Finally, it is noted that the average shortest path length did not give distinct differences at the same extent as the average degree (results not shown here).

## 4. Conclusions

The first main conclusion of this study is that different methods for constructing binary networks from weighted networks could detect similarly the differences in correlation structure at the states before the epileptiform discharges (ED), during ED, as well as during ED but after TMS is administered. From the five methods for the derivation of binary networks used in the study, FDR using *p*-values from randomization test is proposed as an appropriate method to form binary correlation networks despite the large computational cost. However, this method did not give the largest differences between the comparisons of the state before the ED with the state at ED before and after TMS. Comparing the cross-correlation to the partial cross-correlation, though the partial cross-correlation measure can distinguish direct from indirect correlation, it did not seem to discriminate as well as cross-correlation the state before the epileptiform discharge (preED) from the state during the epileptiform discharge (ED). It should be noted that alternative methods of analysis such as the correlation matrix analysis proposed by Li *et al.* [21] allow the quantitative, time-resolved investigation of synchronization phenomena in multiple time series and warrant further study as methods for the fine characterization of TMS effects on synchronization patterns in epilepsy.

The second interesting observation is that ED occurrence in patients with focal epilepsy tends to be associated with decreased cross-correlation of the scalp recorded EEG [22]. This finding may appear paradoxical as hyperchronicity is traditionally thought to be a defining characteristic of epileptic discharges. However, recent studies challenge this view and suggest that seizure occurrence is associated at least in part with decreased brain synchronizability.

For instance, Schindler *et al.* [23–25] investigated the zero-lag cross-correlation of multichannel intracranial EEG in patients with focal epilepsy and computed the eigenvalue spectrum of the cross correlation matrix. The authors observed that seizure onset and seizure propagation is associated with a significant decrease of the larger eigenvalues indicating decorrelation of the EEG. This finding was consistent from seizure to seizure and occurred irrespective of the anatomical location of seizure onset or the total seizure duration. Accordingly, it appears to be a generic characteristic of focal seizures. The authors ascribed the EEG desynchronization to the different propagation times of locally synchronous ictal discharges from the seizure onset zone to other brain areas. Alternatively and more speculatively they suggested that the intense neuronal firing at seizure onset saturates “hub” neurons which are densely interconnected with other network elements. The silencing of these hub neurons results in the functional disconnection between local substructures and is ultimately reflected in decreased coupling in the EEG [23]. In contrast, seizure termination is associated with increased coupling [24,25]. Interestingly, the increased cross-correlation appears prior to seizure ending raising the possibility that it may represent a self-emergent mechanism for the cessation of epileptic activity. Similarly, we did not observe regaining of connectivity with time after the ED onset and during the ED, but rather by the end of the ED, as there was no systematic upward trend within the 10 sec ED (with no TMS), but rather fluctuations around a level, tending to be lower than the level for the 10 sec just before the ED onset.

Based on this observation, Schindler, Elger and Lehnertz [24,25] suggested that one possible mechanism for terminating epileptic seizures with brain stimulation techniques is the enhancement of brain synchronization. This proposition is in line with our observation that TMS aborted epileptiform discharges and concurrently prevented the ED-associated reduction in cross-correlation thereby restoring brain connectivity to pre-ED levels. It is clear that further studies are warranted in order to clarify whether connectivity changes are causally related to seizure termination or if they are simply an epiphenomenon of the cessation of epileptic activity.

In conclusion, simple time-domain connectivity measures, such as cross-correlation and partial cross-correlation, can detect alterations in the EEG connectivity structure and can be used to investigate the effects exerted by TMS on the correlation network in patients with focal epilepsy.

## Figures and Tables

**Table 1.** The *p*-values of the paired t-test and the AUROC values computed on the average degree for the paired comparisons preED-preTMS and preED-postTMS for the 10 episodes with TMS and preED-ED for the 10 episodes without TMS for the method constructing binary networks, cross-correlation *r_ji_*(*τ*) and partial cross-correlation *ϕ_ji_*(*τ*) for *τ* = 0 and *τ* = 1.

**Method**	**PreED-PostTMS**		**PreED-PreTMS**		**PreED-ED**	

	***p*-Value**	**AUROC**	***p*-Value**	**AUROC**	***p*-Value**	**AUROC**

cross-correlation, lag zero						

P-VALUE	0.502	0.65	0.027	0.68	0.057	0.69
P-VALUE-R	0.749	0.57	0.066	0.70	0.025	0.72
FDR	0.487	0.66	0.021	0.72	0.032	0.70
FDR-R	0.820	0.57	0.073	0.72	0.018	0.73
THRESH	0.203	0.69	0.042	0.74	0.090	0.70

cross-correlation, lag one						

P-VALUE	0.335	0.68	0.039	0.71	0.222	0.58
P-VALUE-R	0.445	0.60	0.077	0.71	0.122	0.58
FDR	0.336	0.68	0.028	0.72	0.149	0.58
FDR-R	0.241	0.62	0.089	0.67	0.127	0.55
THRESH	0.110	0.69	0.028	0.70	0.150	0.66

partial cross-correlation, lag zero						

P-VALUE	0.634	0.59	0.044	0.68	0.043	0.71
P-VALUE-R	0.943	0.54	0.254	0.62	0.013	0.72
FDR	0.993	0.58	0.042	0.72	0.010	0.73
FDR-R	0.622	0.52	0.836	0.58	0.474	0.62
THRESH	0.646	0.65	0.271	0.67	0.047	0.75

partial cross-correlation, lag one						

P-VALUE	0.358	0.64	0.063	0.68	0.055	0.62
P-VALUE-R	0.069	0.69	0.231	0.62	0.117	0.58
FDR	0.220	0.69	0.053	0.65	0.040	0.59
FDR-R	0.117	0.63	0.626	0.64	0.172	0.69
THRESH	0.549	0.68	0.331	0.69	0.062	0.76
